# Secreted Protein Acidic and Rich in Cysteine Is a Matrix Scavenger Chaperone

**DOI:** 10.1371/journal.pone.0023880

**Published:** 2011-09-16

**Authors:** Alexandre Chlenski, Lisa J. Guerrero, Helen R. Salwen, Qiwei Yang, Yufeng Tian, Andres Morales La Madrid, Salida Mirzoeva, Patrice G. Bouyer, David Xu, Matthew Walker, Susan L. Cohn

**Affiliations:** 1 Department of Pediatrics, University of Chicago, Chicago, Illinois, United States of America; 2 Department of Pathology, Northwestern University, Chicago, Illinois, United States of America; 3 Department of Surgery, University of Chicago, Chicago, Illinois, United States of America; 4 Committee on Cancer Biology, University of Chicago, Chicago, Illinois, United States of America; University of Arkansas for Medical Sciences, United States of America

## Abstract

Secreted Protein Acidic and Rich in Cysteine (SPARC) is one of the major non-structural proteins of the extracellular matrix (ECM) in remodeling tissues. The functional significance of SPARC is emphasized by its origin in the first multicellular organisms and its high degree of evolutionary conservation. Although SPARC has been shown to act as a critical modulator of ECM remodeling with profound effects on tissue physiology and architecture, no plausible molecular mechanism of its action has been proposed. In the present study, we demonstrate that SPARC mediates the disassembly and degradation of ECM networks by functioning as a matricellular chaperone. While it has low affinity to its targets inside the cells where the Ca^2+^ concentrations are low, high extracellular concentrations of Ca^2+^ activate binding to multiple ECM proteins, including collagens. We demonstrated that *in vitro*, this leads to the inhibition of collagen I fibrillogenesis and disassembly of pre-formed collagen I fibrils by SPARC at high Ca^2+^ concentrations. In cell culture, exogenous SPARC was internalized by the fibroblast cells in a time- and concentration-dependent manner. Pulse-chase assay further revealed that internalized SPARC is quickly released outside the cell, demonstrating that SPARC shuttles between the cell and ECM. Fluorescently labeled collagen I, fibronectin, vitronectin, and laminin were co-internalized with SPARC by fibroblasts, and semi-quantitative Western blot showed that SPARC mediates internalization of collagen I. Using a novel 3-dimentional model of fluorescent ECM networks pre-deposited by live fibroblasts, we demonstrated that degradation of ECM depends on the chaperone activity of SPARC. These results indicate that SPARC may represent a new class of scavenger chaperones, which mediate ECM degradation, remodeling and repair by disassembling ECM networks and shuttling ECM proteins into the cell. Further understanding of this mechanism may provide insight into the pathogenesis of matrix-associated disorders and lead to the novel treatment strategies.

## Introduction

SPARC is a matricellular protein with multiple functions. It regulates the assembly and organization of the extracellular matrix (ECM) [1], modulates multiple intracellular signaling pathways and affects cell migration, proliferation and differentiation [2]. SPARC expression is tightly regulated to confine its activity to the sites of matrix remodeling. It binds multiple structural and soluble ECM proteins in a Ca^2+^-dependent manner, including collagens [3], [4], [5], [6], vitronectin [7], fragments of fibrinogen [8], thrombospondin-1 [9], [10], vascular endothelial growth factor (VEGF) [11] and platelet-derived growth factor (PDGF) [12]. Although the functional significance of this binding is unclear, it has been suggested that SPARC may function as an extracellular chaperone [13]. SPARC is a secreted protein that is induced by heat shock and other forms of stress [14], [15], and its secondary structure remains stable up to 50°C [16]. In a standard assay for chaperone activity, SPARC prevents thermal aggregation of alcohol dehydrogenase (ADH) [16]. It has also been demonstrated to function as a collagen chaperone by affecting the formation of fibrils *in vitro* [17].

SPARC is a major non-structural protein in bone, where extensive matrix remodeling is required to maintain its composition. During bone remodeling, osteoclasts mediate scavenging of collagen through an endosomal/lysosomal pathway with subsequent degradation or removal into the bloodstream by transcytosis [18]. After limited proteolysis by cathepsin K, SPARC is also internalized by osteoclasts and their precursors, mononuclear cells [19]. Internalization of SPARC has been described in activated macrophages and sinusoidal endothelial cells [20] and embryonic chicken cells [21]. SPARC-like peptides are internalized by integrin α5β1 complex in adipose stem cells [22]. We previously demonstrated that mouse NIH/3T3 fibroblasts internalize exogenously added SPARC [23].

So far, no functional model which integrates binding to matrix proteins, chaperone activity, internalization, and other properties of SPARC has been proposed. In this study, we demonstrated that SPARC acts as a scavenger chaperone, which mediates degradation of ECM. Binding of SPARC to the matrix proteins is regulated by a Ca^2+^-switch, and leads to the disassembly of ECM networks. We showed that SPARC shuttles between the intra- and extracellular space and directs internalization of ECM proteins. Further, using fluorescent ECM networks deposited by live cells, we demonstrated that this chaperone activity is required for matrix degradation.

## Materials and Methods

### Animals, cell lines and reagents

SPARC-null mice were obtained from Dr. Helene Sage (Benaroya Research Institute, Seatle, WA) and bred into 129X1/SVJ strain for 3 generations. Animals were kept in a pathogen-free facility and treated according to the NIH guidelines for animal care and use. Protocols were approved by the Animal Care and Use Committee at The University of Chicago, approval 71829. To isolate primary fibroblasts from the skin of SPARC-null and wild type mice, tissues were rinsed with sterile phosphate buffered saline (PBS), sectioned, and placed in DMEM media (Life Technologies Inc, Grand Island, NY) supplemented with 10% fetal bovine serum (FBS, Life Technologies). Fibroblasts were allowed to spread at 37°C in 5% CO_2_, tissue sections were removed and cell morphology was confirmed. All primary cultures were maintained for no longer than 6 passages. Primary human skin fibroblasts BJ, NIH/3T3 mouse fibroblasts, and HEK293 cells were purchased from the American Type Culture Collection (ATCC, Manassas, VA) and cultured at 5% CO_2_ in DMEM with 10% FBS. Recombinant histidine-tagged SPARC was produced in HEK293 cells and purified as described [24].

### Collagen fibril assays

For collagen fibril formation [25], 33 µl of acidic solution of bovine collagen I (Vitrogen, Palo Alto, CA) was neutralized and diluted to 1 mg/ml with 66 µl of PBS, containing indicated amounts of SPARC or BSA (Sigma, St. Louis, MO) at 4°C in a 96-well plate. Fibrillogenesis was started by placing the samples in a Synergy 2 plate reader (BioTek, Winooski, VT) pre-warmed to 37°C. Turbidity was monitored by measuring absorbance at 360 nm every 2 min for 2 h at 37°C. For fibril degradation, collagen gels were pre-formed in 96-well plates as above. Indicated amounts of SPARC or BSA were added to the collagen gels in 30 µl of PBS at room temperature. Where indicated, Ca^2+^ or EDTA was added to the protein solutions to achieve 1 mM final concentrations. Fibril degradation was started by placing samples in a plate reader pre-warmed to 47°C and monitored by recording the turbidity at 360 nm every 2 min for 100 min. All data were analyzed by subtracting blank readings and calculating the average of triplicate measurements in at least three experiments. Statistical significance was determined with single-factor ANOVA.

### Immunofluorescence

Cultured cells grown on coverslips were fixed with 1% paraformaldehyde at room temperature for 20 min, washed twice with PBS and permeabilized with 70% ethanol at −20°C for at least 20 min. Non-specific immunostaining was blocked by pre-incubation in PBS with 5% donkey serum. Target protein expression was detected by incubation in blocking solution for 1 h at room temperature with goat anti-SPARC antibody (R&D, Minneapolis, MN) at 1∶200 dilution, and mouse anti-dynamin antibody (Merck, Darmstadt, Germany) at 1∶200 dilution. Immunocomplexes were visualized with FITC-labeled anti-goat and Texas Red-labeled anti-mouse secondary antibodies (Jackson Immunoresearch Labs, West Grove, PA). Slides were mounted in ProLong Gold (Invitrogen, Carlsbad, CA) and examined under a DB IRB inverted fluorescent microscope (Leica, Heidelberg, Germany).

### Fluorescent matrix assays

Green fluorescent tags were added to recombinant SPARC or collagen I using Alexa Fluor 488 Microscale Labeling Kit (AF488, Invitrogen, Carlsbad, CA), and red fluorescent tags were added to collagen I, fibronectin (Millipore, Billerica, MA), laminin (Chemicon, Temecula, CA), and vitronectin (Promega, Madison, WI) with Alexa Fluor 594 Microscale Labeling Kit (AF594, Invitrogen, Carlsbad, CA) using manufacturer's protocol. In most experiments, collagen I was denatured by boiling for 5 min prior to labeling. This treatment prevents formation of fibrils in the labeling reaction and significantly enhances its efficiency. Boiled collagen was functionally indistinguishable from native collagen in any of the experiments in this study.

For the treatment with soluble labeled ECM components and SPARC, fibroblasts were cultured overnight on glass coverslips in 24-well plates. Fluorescently labeled proteins were added to the fibroblasts at 1 µg/ml in serum-free media as detailed in the Results section for each particular experiment. After the treatment, cells were fixed in 1% paraformaldehyde for 20 min at room temperature, rinsed twice with PBS and mounted in ProLong Gold. Distribution of labeled proteins was examined under a Leica DB IRB fluorescent microscope.

Three-dimensional matrixes were pre-deposited using published methodology [26], with added fluorescent proteins to allow visualization. Mouse NIH/3T3 fibroblasts were cultured on Matrigel (Discovery Labware, Bedford, MA)-coated coverslips in the presence of 5 µg/ml of AF488-labeled collagen I alone or in combination with 5 µg/ml of AF-594-labeled fibronectin in complete media. When dense fluorescent networks were visible by fluorescent microscopy, cells were removed by treatment with 20 mM NH_4_OH, coverslips were rinsed twice with PBS, and denuded matrixes were stored in PBS at 4°C for up to 2 weeks. To assess matrix remodeling and degradation, SPARC-null and wild type fibroblasts were cultured on fluorescent matrixes for the indicated periods, fixed and mounted as above. Matrixes were examined under a Leica DB IRB microscope and photographed. The matrix area was quantified at ×600 magnification in at least quadruplicate fields in three independent experiments using ImagePro software. Statistical significance was determined with Student's t-test for individual time points.

### Internalization and pulse-chase assays

Primary SPARC-null fibroblasts were cultured overnight in a 24-well plate in complete media, rinsed with PBS and treated with proteins in serum-free media. To assess the internalization of SPARC, cells were treated with 5 µg/ml or 1 µg/ml of recombinant SPARC. After indicated periods, cells were rinsed with PBS, lysates were prepared and intracellular proteins detected by Western blot analysis as described [27] using anti-SPARC antibody at 1∶1000 dilution.

To study the effect of SPARC on the internalization of collagen, cells were treated with 5 µg/ml of SPARC and 5 µg/ml of AF488-labeled collagen I alone or in combination. After indicated periods, cells were rinsed with PBS and lysed with 2% sodium deoxycholate in PBS. Intracellular proteins were detected by Western blot analysis as above with anti-AF488 antibody (Invitrogen, Carlsbad, CA) at 1∶5000 dilution.

For the pulse-chase assay, SPARC-null fibroblasts were treated with 1 µg/ml of recombinant SPARC for 2 h in serum-free DMEM. After the pulse, exogenous SPARC was removed by rinsing with PBS and replacing the media. The amount of internalized SPARC in cell lysates and excreted SPARC in the media was determined by Western blot as above. All experiments were repeated at least three times with consistent results. For quantitative analysis, densities in individual experiments were normalized and represented as an average of three experiments ±SD. Statistical significance for individual time points was determined with Student's t-test.

## Results

### Disassembly of collagen networks by SPARC is regulated by a Ca^2+^-switch

**Figure 1:**
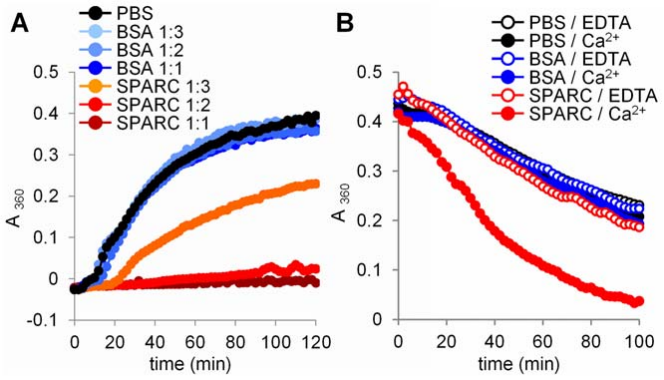
SPARC mediates disassembly of ECM networks. (A) Collagen fibril formation at 37°C was almost completely prevented by SPARC. Density of collagen networks measured by opalescence at 2 h was decreased by 99.7±7.2% (F = 249) for 1∶1 and by 91.8±8.2% (F = 224) for 1∶2 molar ratio of SPARC to collagen monomer respectively, as compared with PBS control. At 1∶3 ratio, SPARC prevented formation of collagen fibrils by 44.9±14.2% (F = 46). BSA at the same ratios did not cause statistically significant change in fibril formation (F<3.92). (B) Degradation of pre-formed collagen fibrils at 47°C doubled when SPARC was addedat 1∶1 molar ratio to collagen monomer with the complete loss of opacityin 100 min. Collagen fibril degradation was only enhanced by SPARC in 1 mM Ca^2+^ (F = 60), and was completely abolished in 1 mM EDTA (F<3.90). The same amount of BSA or PBS without proteins did not affect degradation of collagen fibrils neither in Ca^2+^, nor EDTA (F<3.90).

SPARC is a secreted stress-induced protein with chaperone properties [14], [15]. Chaperones of the heat-shock family assist in the folding and repair of cytoplasmic proteins or facilitate their unfolding and degradation [28]. Because folding requires energy and ATP is lacking outside the cell, we hypothesized that SPARC mediates the unfolding of target proteins. To test this hypothesis, the effect of SPARC on the formation of collagen fibrils [25] was examined. As shown in figure 1a, the addition of SPARC to collagen monomers at 1∶1 molar ratio inhibited the formation of collagen fibrils by 99.7%. The effect was concentration-dependent, and at a 1∶2 or 1∶3 ratio per collagen monomer, SPARC inhibited formation of fibrils by 91.8% and 44.9% respectively, demonstrating that SPARC prevents formation of collagen fibrils.

To directly test the effect of SPARC on the disassembly of collagen networks, pre-formed collagen fibrils were incubated at 47°C, a temperature that induces fibril degradation and a loss of opacity. The addition of SPARC at 1∶1 ratio per collagen monomer caused complete loss of opacity in 100 min, a nearly a 100% increase in fibril degradation compared to control (figure 1b). These results indicate that SPARC mediates the disassembly of collagen networks.

Ca^2+^ affinity of SPARC EF-hand domains [29] suggests that they may be able to function as a sensor, discriminating between low intracellular and high extracellular concentrations of Ca^2+^. We have tested if the ability of SPARC to disassemble collagen fibrils requires high concentrations of Ca^2+^. As shown in figure 1b, this activity was completely lost in the presence of EDTA, demonstrating that Ca^2+^ binding to the EF-hand domains regulates chaperone activity of SPARC, switching between inactive intracellular and active extracellular conformations.

### SPARC shuttles between extra- and intracellular compartments

**Figure 2:**
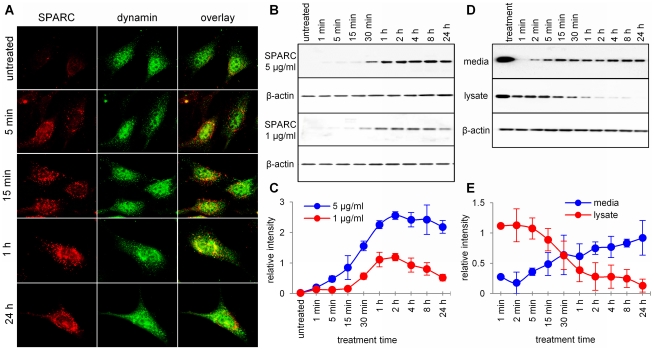
Internalization of SPARC by fibroblastcells. (A) After treatment with exogenous SPARC for indicated periods, bright intracellular vesicles containing internalized SPARC were detected by immunofluorescence in the cytoplasm of mouse NIH/3T3 fibroblasts. Exposure time was chosen to minimize interferenceof endogenously expressed SPARC, which can be detected in untreated cells. Lack of co-localization with the marker of caveolae and clathrin-coatedvesicles, dynamin, was apparent at 5 and 15 min time points. (B) Representative Western blot and (C) semi-quantitative analysisof SPARC in the cytoplasm of primary fibroblasts, isolated from SPARC-nullmice. After addition of 5 µg/ml of SPARC tothe culture media, it was detected in cell lysates in 1 min with increasing accumulation until 1–2 hours. Treatment with 1 µg/ml of SPARC resulted in weaker internalization which followed the same dynamic. (D) Representative Western blot and (E) semi-quantitative analysis of SPARC turnover in the pulse-chase assay. SPARC-null fibroblasts were treated with 5 µg/ml of SPARC added to themedia. The amount of SPARC in the media and internalized bythe fibroblasts at the end of pulse treatment is shown in the first lane.After SPARC was removed from the media, both media and cell lysates were collected at the indicated time points. Western blot analysis showed rapid, time-dependent release of SPARC from the fibroblasts, accompanied bya decrease in the amount of intracellular SPARC.

The ability of SPARC to bind multiple extracellular proteins [2] suggests that after its secretion and activation by Ca^2+^, the majority of SPARC is bound to various ECM proteins. Internalization of secreted SPARC by different cell types has been previously described [19], [20], [21], [23]. We confirmed that exogenously added SPARC is actively internalized by all types of fibroblasts, including mouse NIH/3T3 fibroblasts, human primary skin fibroblasts BJ and fibroblasts isolated from SPARC-null mice (data not shown). In mouse NIH/3T3 fibroblasts, intracellular SPARC-positive vesicles did not undergo visible degradation for as long as the cells remained alive in serum-free media, and did not appear to co-localize with dynamin, the marker of caveolae and clathrin-coated vesicles (figure 2a).

SPARC added to the media can be detected by Western blot inside primary fibroblasts from SPARC-null mice as early as in 1 min. Intracellular SPARC levels increased up to 1–2 hours after the start of treatment and remained relatively constant afterwards (figure 2b and c). After adding exogenous protein to the SPARC-null fibroblasts in a 2 h pulse, internalized SPARC can be detected in the fresh media 1 min after the pulse. The amount of released protein stably increased over a period of 24 h (figure 2d and e), which correlated with a corresponding decrease in the cytoplasmic SPARC.

Together, these experiments demonstrate that secreted SPARC is rapidly internalized by the fibroblasts, and rapidly released back to the extracellular space, indicating that SPARC shuttles between the extracellular matrix and the cytoplasm. It is therefore a matricellular chaperone, in full accordance with the meaning of term “matricellular” [30].

### SPARC does not affect deposition of ECM networks

**Figure 3:**
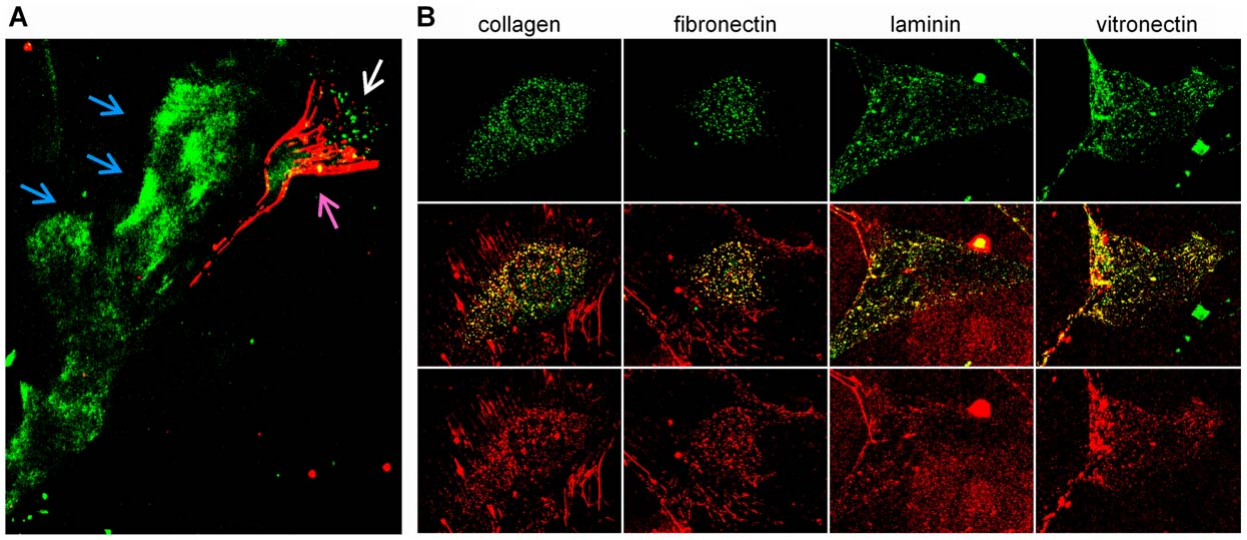
Co-internalization of SPARC and ECM proteins in fibroblasts. (A) Primary mouseSPARC-null fibroblasts were attached to glass coverslips overnight and treated with red AF594-labeled collagen and green AF488-labeled SPARC. After 1 h of treatment, strong fibrillar collagennetworks were deposited at the trailing edge of a moving fibroblast (purple arrow). Fluorescent SPARC bound ECM tracks, deposited along the movement path (blue arrows) and was internalized at the leading edge of the fibroblast (white arrow). (B) SPARC-null fibroblasts were plated and treated with green AF488-labeled SPARC and red AF594-labeled ECM proteins as above. Allmatrix proteins were deposited into apparently normal extracellular networks and internalized by the cells. Strong co-localizationof all internalized ECM proteins with SPARC is evident by the yellow overlap of the two colors.

To study the impact of SPARC on the formation of ECM networks by live cells, SPARC was conjugated with green fluorescent dye AF488, and ECM proteins were conjugated with red fluorescent dye AF594. When added to the cultured primary fibroblasts isolated from SPARC-null mice, red fluorescent collagen was deposited into extracellular fibrillar networks in a pattern consistent with its normal function. In a moving fibroblast, the majority of collagen was deposited in the fibrils at the trailing edge, while labeled SPARC was actively internalized at the leading edge. SPARC also bound ECM components deposited by the fibroblast along the moving path, visible as green tracks left behind the cell (Figure 3a). Quiescent cells also deposited labeled collagen into extracellular fibrillar networks. In addition, significant amounts of collagen were found in cytoplasmic vesicles, indicating that collagen is actively internalized. Labeled SPARC was also internalized and overlay of red and green colors in figure 3b shows that both proteins are contained in the same vesicles. Labeled with red AF594 fibronectin, laminin and vitronectin were also deposited into extracellular networks by primary fibroblasts and co-internalized with SPARC in the same vesicles.

**Figure 4:**
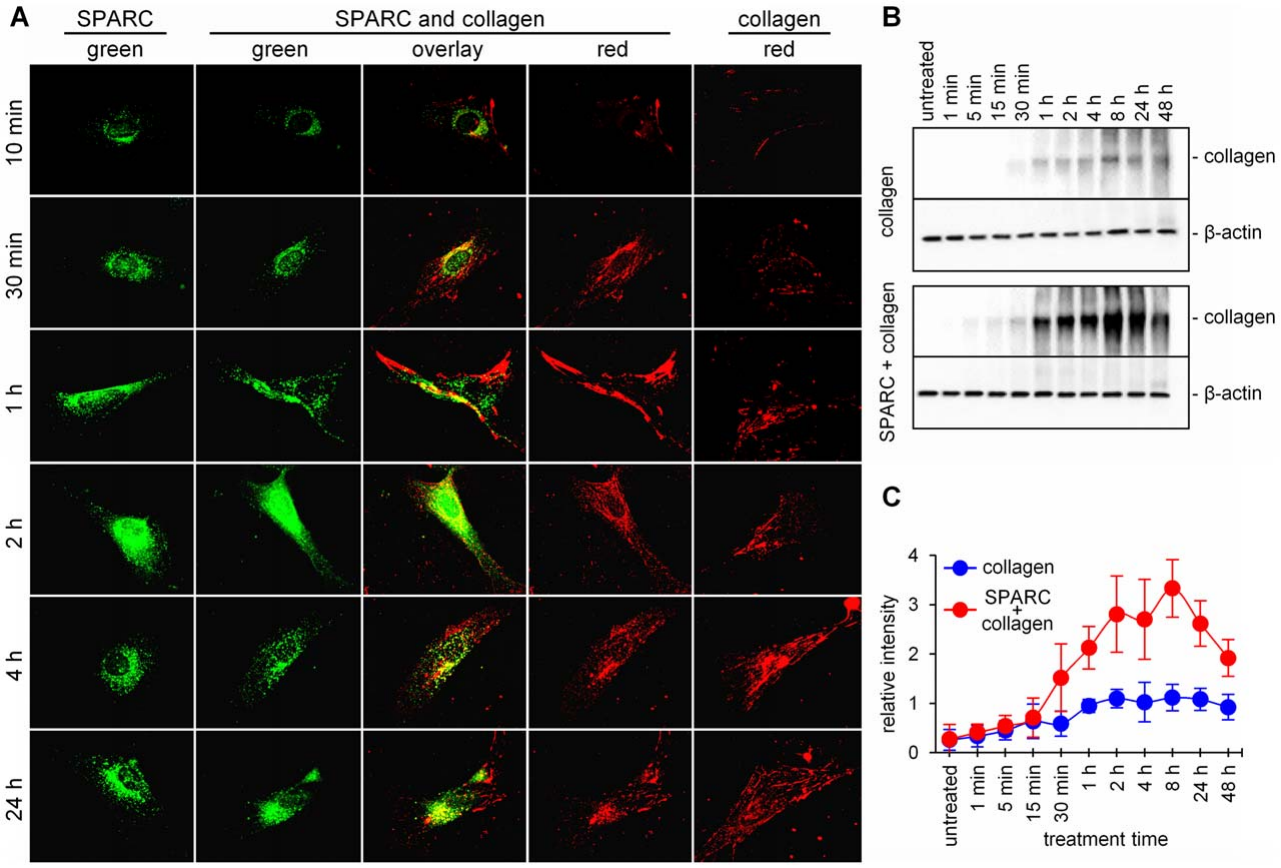
Effect of SPARC on collagen turnover. (A) SPARC-null primary mouse fibroblasts were treated with green AF488-labeled SPARC (left panel), red AF594-labeled collagen (right panel) or their combination (middle panels). Formation of cytoplasmic vesicles containing internalized SPARC follows the same dynamic, as determined by Western blot analysis. Collagenwas deposited into fibrillar extracellular networks in 2–4 hafter the treatment. When significant uptake of collagen was observed, it was internalized in SPARC-positive vesicles. (B) Representative Western blot and (C) semi-quantitative analysis of exogenously added AF488-labeled collagen in the cytoplasm of SPARC-null fibroblasts. Treatment with SPARC led to a statistically significant (p<0.05) 2–3 times increase in collagen internalization at 30 min–48 h. Substantial degradation of collagen was also be observed in 24 and 48 h after the treatment.

The dynamics of the assembly of collagen networks suggested that their formation occurs exclusively outside of the cell and does not require intracellular trafficking of collagen or the presence of SPARC (figure 4a and b). First extracellular fibrils form as quickly as 10 min after the addition of fluorescent collagen and the networks continue to expand until several hours after the treatment (figure 4a). Internalization of collagen starts only when the first fibrils are formed, and it is accompanied by the degradation of the networks, which becomes apparent at 24 h after the treatment.

### SPARC promotes internalization of collagen

Binding SPARC to the ECM proteins outside the cell and the co-localization of SPARC and ECM proteins inside the cell suggests that SPARC directs the internalization of its targets. To test this hypothesis, SPARC-null fibroblasts were treated with collagen alone and collagen in combination with SPARC. Exogenously added collagen was labeled with AF488 to distinguish it from endogenously produced collagen. Using anti-AF488 antibody, we showed that internalization of collagen occurs immediately after the treatment and continues to increase for 2–4 h (figure 4b and c). Statistically significant increase of collagen internalization by SPARC was first observed in 30 min after the treatment (p = 0.005), at the same time when robust extracellular networks are formed (figure 4a). In 2–4 h after the treatment, the amount of internalized collagen was increased by SPARC ∼3 fold. Together with previous observations, this indicates that SPARC mediates the degradation of pre-formed ECM networks and directs internalization of their components.

### SPARC promotes degradation of ECM networks

**Figure 5:**
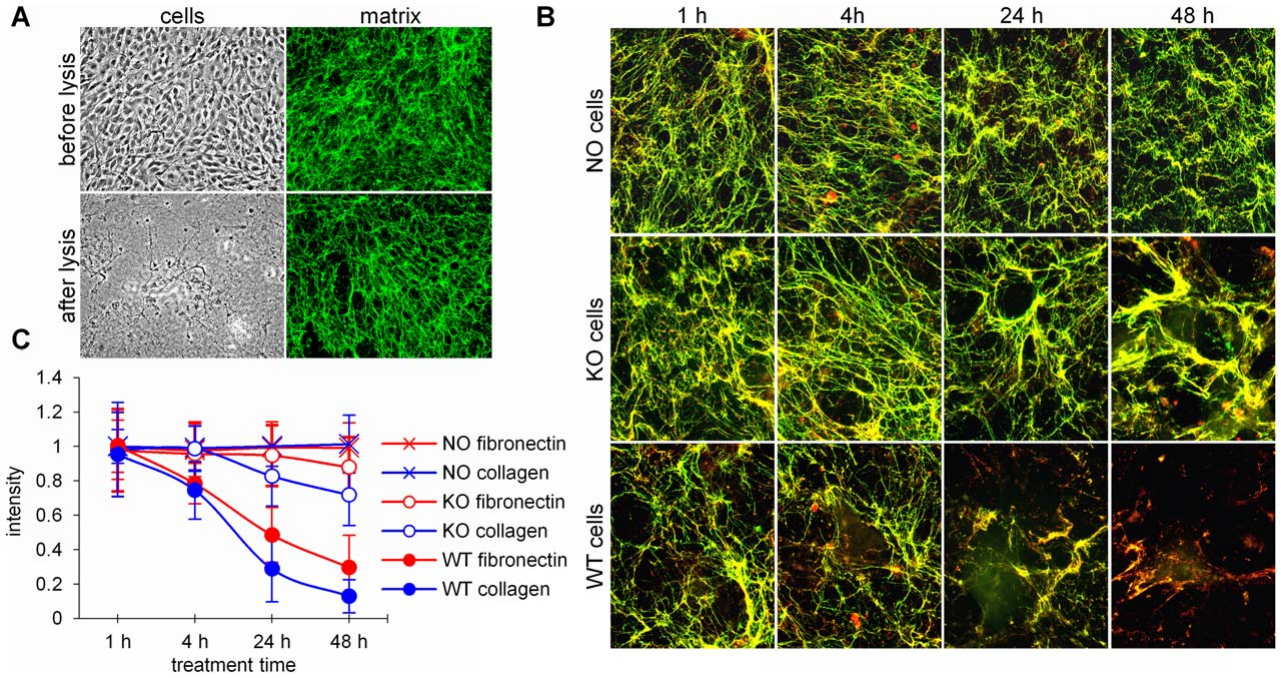
Degradation of fluorescent ECM networks by SPARC. (A) Deposition of fluorescently labeled ECM. NIH/3T3 fibroblasts were grown on Matrigel-covered coverslips in the presence of green AF488-labeled collagen. Fluorescent collagen was incorporated into the normal 3-dimentional ECM networks, deposited by live cells. After cells were removedby mild lysis, the fluorescently labeled matrix remained intact. (B) Networks were formed by NIH3T3 cells with green AF488-labeled collagenand red AF594-labeled fibronectin. After cell removal (NO cells), fluorescent networks remained intact in tissue culture conditions for at least 48 hours. SPARC knock-out fibroblasts (KO cells) grown on pre-formed fluorescent matrix re-arrangedthe networks by bending, merging and thickening fibrils around the cells. Wild type cells (WT cells) also remodeled the fibrils and caused matrix degradation, which was almost complete in 48 h. (C) Quantitative image analysis of the above experiment with separation of red (fibronectin) and green (collagen I) colors. Measured by the loss of fluorescence, matrix degradation was statistically significant (p<0.05) starting at 2 h after the attachment of the wild type cells (WT), compared with untreated matrices (NO). SPARC knock-out fibroblasts (KO) did not cause statistically significant decreasein fluorescence (p>0.05).

To directly test the role of SPARC in the degradation of ECM networks, we modified an experimental model of 3-dimentional matrixes *in vitro* [26] by adding fluorescently labeled ECM proteins. Figure 5a demonstrates that cultured NIH/3T3 fibroblasts form extensive 3-dimentional ECM networks, which incorporate fluorescent collagen, and remain intact after removal of the cells.

To study ECM degradation, the networks were formed with green AF488-labeled collagen and red AF594-labeled fibronectin. Both proteins showed almost complete co-localization, the networks remained intact after cell removal and were stable in tissue culture conditions for at least 48 h (figure 5b). When SPARC-null fibroblasts were grown on these pre-deposited fluorescent matrices, re-arrangement of fibrils was detected shortly after cell attachment, about 4 h after plating: fibrils bent around the cells, merged and increased in thickness. Although some degradation of fibrils occurred after 24–48 h, the loss of fluorescence by quantitative image analysis was not statistically significant. In contrast, when SPARC-positive wild type fibroblasts were grown on these matrices, the re-arrangement of fibrillar networks was accompanied by intense degradation. The loss of fluorescence was statistically significant at 6 h and almost complete at 24–48 h, demonstrating that SPARC mediates degradation of ECM networks, acting as a scavenger chaperone.

## Discussion

Recent discoveries started to change the traditional perception of ECM as an inert support for live cells. Early embryonic lethality in knockout animals proved that matrix proteins are essential for the development and architecture of tissues [31]. The discovery of integrins and cell-matrix signaling demonstrated that ECM controls the most critical aspects of cell physiology and maintains the “integrity” of the tissue [32]. Advances in stem cell research uncovered significant potential for regeneration and repair of abnormalities in the cellular component, further emphasizing the importance of extracellular abnormalities. Matrix abnormalities have already been shown to cause a number of chronic and acute diseases, age-associated and genetic disorders [33], and to support cancer progression [34]. Understanding the mechanism of matrix remodeling and repair is essential for disease prevention and the development of new therapies.

SPARC is a non-structural ECM protein associated with matrix remodeling, which appears in the first multicellular organisms. It remains highly conserved during evolution, suggesting the importance of its function. In higher metazoans, this function has been further multiplied in a number of structurally and functionally related proteins [35], [36], [37]. Lethal in primitive organisms, abrogation of SPARC has progressively milder effects in higher animals due to this functional compensation [1]. In more than 30 years since its discovery [38], SPARC has been shown to act as an essential modulator of cell-matrix interactions, which affects cell attachment, migration, proliferation, differentiation, signaling, matrix deposition and degradation [2]. Nevertheless, the molecular mechanism responsible for these functions of SPARC remains unclear.

In this study we demonstrate that SPARC functions as an extracellular chaperone that mediates the disassembly and degradation of ECM networks. We propose that at high concentrations of Ca^2+^ in the extracellular space, SPARC binds multiple ECM targets, promotes the disassembly of solid networks, and directs the internalization of their components. At low intracellular concentrations of Ca^2+^ it has to release its target, and free SPARC is recycled outside the cell, serving as a shuttle for scavenging various structural and soluble extracellular proteins. Thus, SPARC may represent a new functional class of scavenger chaperones, necessary for the dynamic regulation of ECM composition and the repair of matrix abnormalities.

The chaperone activity of SPARC may be regulated by several factors. Binding SPARC to its known soluble and structural ECM targets is Ca^2+^-dependent [3], [4], [5], [7], [10], [35], [39]. The C-terminal extracellular calcium-binding domain of SPARC contains a canonical pair of EF-hand motifs that cooperatively bind Ca^2+^ with macroscopic K_d_ of 490 nM and 57 nM, and half-saturation at a free Ca^2+^ concentration of 170 nM [29]. These highly conserved helix-loop-helix Ca^2+-^binding motifs are usually found in cytosolic proteins of the Ca^2+^ second messenger system, where they distinguish between intracellular Ca^2+^ concentrations of ∼0.1 µM in resting, and ∼10 µM in activated cells. The regulatory role of EF-hand motifs in secreted proteins is unclear, because extracellular concentrations of Ca^2+^ remain at a constant high level of ∼1.2 mM [29].

High extracellular concentrations of Ca^2+^ are sufficient to bind the EF-hand motifs in SPARC and to induce conformational changes which activate binding to collagen with high affinity [40]. Cytosolic Ca^2+^ concentrations in the resting cell are too low to saturate EF-hand motifs. Although Ca^2+^ concentrations are higher in activated cell or in early endosomes [41], [42], they are insufficient to induce Ca^2+^-dependent conformational transition [43], and SPARC remains in an inactive state with low affinity to its ligands [35]. Thus, the chaperone activity of SPARC may be controlled by a Ca^2+^-switch between a high-affinity ligand-bound state outside and a low-affinity ligand-free state inside the cell.

Cleavage by extracellular proteases increases SPARC affinity to collagens up to 20-fold [5], [44] and may be another important switch in the regulation of its activity. Concentrations of SPARC in tissues are difficult to measure, but micromolar K_d_ for most of the collagens indicate that there may be not enough SPARC for binding in normal conditions. Higher levels of expression and/or activation by extracellular proteases at the sites of tissue remodeling may be necessary to increase binding and to promote scavenging of a wider range of targets.

Knowledge of the range of SPARC targets and affinity of their binding may be important for other aspects of tissue physiology. Although binding of SPARC to serum albumin has not been studied in detail since its discovery [45], this property of SPARC has been ascribed to the mechanism of drug delivery by nanoparticle albumin-bound drugs [46]. It will be important to clarify, whether internalization of SPARC is mediated by the albumin receptor gp60, which has already been shown to bind SPARC on endothelial cells [47], and if the chaperone function of SPARC is required for albumin transcytosis across the endothelial cell layer in normal blood vessels [48], which is stimulated by SPARC [49].

In an apparent contradiction, the phenotype of SPARC knockout mice [50], [51] suggests abnormality in matrix deposition and maturation rather than impaired matrix degradation. These animals develop early-onset cataracts [50], [51], profound age-progressive osteopenia [52], abnormalities in collagen deposition in fragile skin [53], and enlarged adipose tissue [54]. Despite significant acceleration of wound healing [55], scars are abnormal and collagen deposition is impaired in SPARC-null animals with cardiac infarcts, causing increased incidence of cardiac rupture and 4-times higher mortality [56].

The issue can be resolved if proper scavenging is necessary to remove abnormalities during matrix deposition and to prevent premature termination of growing ECM networks. Indeed, unusually high rates of matrix degradation indicate that it is essential for normal ECM deposition and maturation. Most of newly synthesized collagen is immediately degraded, especially in tissues with high functional requirements. This process increases with age, reaching about a 90% ratio of degradation to synthesis in heart and muscle tissues [57]. A lack of repair may be evident in the eye lens of SPARC-null mice, where accumulated abnormalities lead to a loss of transparency. In transgenic animals, SPARC has also been demonstrated to associate with other age- and matrix-related disorders, including periodontosis [58] and decrease in myocardial stiffness [59].

Essentially two mechanisms of collagen degradation are currently recognized. In the extracellular pathway, matrix is cleaved by secreted matrix metalloproteinases and cathepsin [60]. In the intracellular pathway, collagen receptors α_2_β_1_-integrin or urokinase plasminogen activator receptor-associated protein (uPARP/endo180) mediate phagocytosis and lysosomal degradation of collagen in fibroblast cells [61], [62]. Whether SPARC is capable of connecting extra- and intracellular pathways by clearing proteolysed ECM debris, should be addressed in the future. Further studies of dynamic interactions between cells and their extracellular environment will also be required to validate the importance of scavenger chaperone(s) in normal and pathologic physiology. It is nevertheless evident that matrix remodeling and repair is indispensable for the normal function of tissues and organs. A further understanding of SPARC's role as a chaperone for ECM proteins will provide insight into the pathogenesis of matrix-associated disorders, and may lead to the development of novel treatment strategies.
